# The VetMAX™ *M. tuberculosis* complex PCR kit detects MTBC DNA in antemortem and postmortem samples from white rhinoceros (*Ceratotherium simum*), African elephants (*Loxodonta africana*) and African buffaloes (*Syncerus caffer*)

**DOI:** 10.1186/s12917-020-02438-9

**Published:** 2020-06-29

**Authors:** Wynand J. Goosen, Tanya J. Kerr, Léanie Kleynhans, Peter Buss, David Cooper, Robin M. Warren, Paul D. van Helden, Björn Schröder, Sven D. C. Parsons, Michele A. Miller

**Affiliations:** 1 DSI-NRF Centre of Excellence for Biomedical Tuberculosis Research, South African Medical Research Council Centre for Tuberculosis Research, Division of Molecular Biology and Human Genetics, Faculty of Medicine and Health Sciences, Stellenbosch University, P.O. Box 241, Cape Town, 8000 South Africa; 2 Veterinary Wildlife Services, South African National Parks, Kruger National Park, Limpopo, South Africa; 3 Enzemvelo KZN Wildlife, P.O. Box 25, Mtubatuba, 3935 South Africa; 4 Thermo Fisher Scientific; Prionics AG, Wagistrasse 27A; Schlieren, Zurich, Switzerland

**Keywords:** African buffaloes, African elephants, *Mycobacterium bovis*, *Mycobacterium tuberculosis*, PCR, VetMAX™ MTBC detection kit qPCR, white rhinoceros

## Abstract

**Background:**

Bovine tuberculosis and tuberculosis are chronic infectious diseases caused by the *Mycobacterium tuberculosis* complex members, *Mycobacterium bovis* and *Mycobacterium tuberculosis*, respectively. Infection with *M. bovis* and *M. tuberculosis* have significant implications for wildlife species management, public health, veterinary disease control, and conservation endeavours.

**Results:**

Here we describe the first use of the VetMAX™ *Mycobacterium tuberculosis* complex (MTBC) DNA quantitative real-time polymerase chain reaction (qPCR) detection kit for African wildlife samples. DNA was extracted from tissues harvested from 48 African buffaloes and MTBC DNA was detected (test-positive) in all 26 *M. bovis* culture-confirmed animals with an additional 12 PCR-positive results in culture-negative buffaloes (originating from an exposed population). Of six MTBC-infected African rhinoceros tested, MTBC DNA was detected in antemortem and postmortem samples from five animals. The PCR was also able to detect MTBC DNA in samples from two African elephants confirmed to have *M. bovis* and *M. tuberculosis* infections (one each). Culture-confirmed uninfected rhinoceros and elephants’ samples tested negative in the PCR assay.

**Conclusions:**

These results suggest this new detection kit is a sensitive screening test for the detection of MTBC-infected African buffaloes, African elephants and white rhinoceros.

## Background

Bovine tuberculosis (bTB) and tuberculosis (TB) are chronic infectious diseases caused by the *Mycobacterium tuberculosis* complex (MTBC) members, *Mycobacterium bovis* (*M. bovis*) and *Mycobacterium tuberculosis* (*M. tuberculosis*), respectively. Many countries in Africa, especially those with a high TB burden, are heavily reliant on agriculture and tourism to support their economy. Increased opportunities for disease transmission between animals and people are observed with growing human settlements into land previously used for agriculture or natural wildlife habitats [1]. In South Africa, African buffaloes (*Syncerus caffer*) are believed to be a key *M. bovis* wildlife maintenance host, with reported spill-over to other wildlife species and cattle [2, 3]. In addition, cases of bTB have also been reported in people in African countries, especially those in contact with animals [4]. Cases of *M. tuberculosis* infection also occur in domestic cattle in rural areas of Africa as well as captive wildlife and pets [5–8].

In 2016, bTB was discovered in several free-ranging white rhinoceroses (*Ceratotherium simum*) and a single black rhinoceros (*Diceros bicornis*) as well as disease associated with *M. tuberculosis* in an African elephant bull in the Kruger National Park (KNP) [7–9]. In South Africa, the largest populations of rhinoceros and elephants reside in KNP, a bTB endemic area. Therefore, infection with *M. bovis* and *M. tuberculosis* have significant implications for species management, public health, veterinary disease control, and conservation endeavours.

Animals infected with MTBC can remain asymptomatic for months or years. Therefore, strategies for management and control require early detection of infection, typically through the measurement of antigen-specific cell-mediated immune (CMI) responses, rather than basing decisions on the presence of disease. Unfortunately, species-specific in vitro diagnostic assays for detection of CMI responses to *M. bovis* and *M. tuberculosis* are either very limited, as in the case of white and black rhinoceros [10], or non-existent yet for African elephants. Therefore, antemortem TB diagnosis in these species still relies on mycobacterial culture of respiratory tract samples such as bronchoalveolar lavage fluid (BALF) from rhinoceros or BALF and trunk washes (TW) from African elephants. Because mycobacterial culture is regarded as an imperfect gold standard [11, 12], detection of mycobacterial DNA in animal samples has been used as an alternative or ancillary diagnostic method [12, 13]. In humans, PCR assays have become a common molecular tool for rapid detection of TB patients, and now can provide a definitive diagnosis [14, 15]. During the 1990’s, numerous PCRs had been developed and used for the direct detection of MTBC members in cattle tissue specimens [13]. In those studies, discrepancies for their sensitivity (63–97%) and specificity (50–97%) were frequently reported between tissue samples with visible and non-visible lesions [13]. It was only after the introduction of the Real-Time quantitative PCR (RT-qPCR) for the detection of MTBC members that sensitivity (74–100%) and specificity (97–100%) values were considerably increased [13]. It was observed that the variability between studies did not only depend on the type of PCR used (conventional, nested or RT-qPCR), but also on the number of PCR DNA targets and reagents, the type and number of specimens included, and the methods used for DNA isolation. To date, the largest reported study for the use of a standardised commercial RT-qPCR in cattle was the use of the VetMAX™ MTBC qPCR Kit and a sensitivity of 87.7% and a specificity of 97% were reported [13]. Since then, this specific RT-qPCR has been used extensively in various wildlife like boars, badgers, and deer and subsequently drastically improved bTB wildlife surveillance programs in France [16–18].

In this study, we describe the use of the VetMAX™ MTBC qPCR Kit for the detection of the insertion element IS*6110* from stored tissue and respiratory tract samples previously collected from African buffaloes, white rhinoceros and African elephants with known MTBC-culture status.

## Results

When tissue samples from the randomly selected buffaloes (*n* = 48) from endemic areas were tested with the VetMAX™ MTBC PCR kit, 38 (79.2%) tested positive (MTBC DNA detected) and 10 (20.8%) tested negative (MTBC DNA not detected). All 26 culture-confirmed *M. bovis*-infected buffaloes were PCR positive (Table 1). Of the 22 culture-negative buffaloes that had been culled based on a positive skin and/or cytokine assay result, 12 were identified as PCR positive (Table 1 and Supplementary Table 1).

**Table 1:** Comparative VetMAX™ PCR Kit test results for tissue and respiratory samples collected from African buffaloes (*n* = 48), white rhinoceros (*n* = 10) and African elephants (*n* = 13) with known mycobacterial culture results

Species	Sample type	No of samples	Mycobacterial culture sample results		VetMax PCR Pos^b^/ MTBC culture Pos samples	VetMax PCR Neg/ MTBC culture Neg samples
			MTBC culture positive (MTBC isolate)^a^	MTBC culture negative		
African buffaloes^c^	Lymph nodes(LNs)	*n* = 47	25 (*M. bovis*)	22	25/25	10/22
	Lung	n = 1	1 (*M. bovis*)	n.a	1/1	n.a
White rhinoceros^d^	LNs	*n* = 23	20 (*M. bovis*)	3	8/20	1/3
	Lung	*n* = 6	6 (*M. bovis*)	n.a	3/6	n.a
	BALF	*n* = 5	n.a	5	n.a	5/5
African elephants^e^	LNs	n = 4	1 (*M. bovis*)	3	1/1	0/3
	Lung	n = 2	1 (*M. tuberculosis*)	1	1/1	0/1
	BALF	n = 13	2 (*M. tuberculosis*)	11	2/2	11/11
	TW	*n* = 12	1 (*M. tuberculosis*)	11	1/1	11/11

^a^Confirmed MTBC culture positive: *M. bovis*- and *M. tuberculosis*-positive by mycobacterial culture and strain typing [19]

^b^VetMAX PCR Kit is a real-time PCR assay that targets the IS*6110* insertion element sequence present in the genome of *M. tuberculosis*, *M. bovis*, *M. africanum*, *M. Microti*, *M. canetti*, *M. caprea* and *M. pinnipedii*

^c^All test-positive African buffaloes were euthanized on either or the single intradermal comparative tuberculin test result and an interferon-gamma (IFN-γ) release assay result

^d^African rhinoceros BALF samples tested were collected from four individual animals suspected of *M. bovis* infection, but not confirmed. One animal had a duplicate BALF sample

^e^African elephant PCR positive/ culture positive BALFs, TW and lung sample were collected from one elephant confirmed to be infected with *Mycobacterium tuberculosis,* one PCR positive/ culture positive LN samples were collected from one *M. bovis* confirmed elephant

Unlike the buffalo, various samples were opportunistically collected from rhinoceros that did not have any previous testing for TB, although they were in a bTB endemic area (KNP). The BALF samples collected from four rhinoceros (animal # 4, 5, 6 and 7) suspected of being *M. bovis*-infected, but unconfirmed by culture, were all PCR negative. Of the six rhinoceros with postmortem samples tested by PCR, MTBC DNA were detected in five animals (Table 1). The 11 tissue samples (lymph nodes and lung) that were *M. bovis* culture-positive from these five rhinoceros were all positive by PCR. An additional two lymph node samples from the same group of *M. bovis*-infected rhinoceros were PCR positive but culture negative. An additional ten tissue samples, collected from four rhinoceros previously identified as *M. bovis*-infected by VetMAX™ qPCR and culture on at least a single sample, were PCR and culture negative. Tissue samples collected from rhinoceros number 10 were PCR negative, but culture positive for *M. bovis* (Supplementary Table 2). All the remaining culture-negative tissue and BAL samples tested were PCR negative (Supplementary Table 2).

Of the 31 antemortem and postmortem samples (6 tissues, 13 BALF and 12 TW) collected, from 13 African elephants, *M. tuberculosis* and *M. bovis* infection was confirmed by mycobacterial culture in 2 elephants; a zoo elephant and 1 free-ranging elephant from a bTB endemic area, respectively (Table 1 and Supplementary Table 2). The VetMAX™ qPCR confirmed infection in all culture-positive samples from these animals. Antemortem samples collected from the *M. tuberculosis*-infected elephant (two BALF and one TW) were all PCR positive, as well as a postmortem lung tissue sample. Only postmortem samples were available from the *M. bovis*-infected elephant. Five lymph node samples were all PCR positive, although only 1 lymph node was culture positive. The remaining 22 antemortem BALF and TW samples collected from 11 apparently healthy immobilized free-ranging elephants were all PCR and culture negative (Table 1 and Supplementary Table 2).

## Discussion

Although the VetMAX™ MTBC qPCR Kit has widely been used for MTBC detection in various wildlife species from all over the world, this study is the first report of its use for detection of MTBC infection in African wildlife species. Courcoul et al. (2014) reported its first use in cattle in France and showed this PCR had better diagnostic performance compared to mycobacterial culture or histopathology, creating the foundation for the use of this PCR kit in livestock [20]. In 2018, improved abattoir bTB surveillance strategies were reported for cattle suspected to be infected with *M. bovis* in France. It was shown that the incorporation of the VetMAX™ MTBC qPCR kit drastically reduced the number of suspected bTB cases raised by false-positive histopathology results and shortening their lock-up periods [21]. Similarly, wildlife national surveillance systems in France were also drastically improved in wildlife species like badgers, wild boar, and deer when using the VetMAX™ MTBC qPCR [16–18]. These findings led to the further evaluation of the VetMAX™ MTBC qPCR kit in African wildlife in this study and showed strong agreement with previously mentioned wildlife studies.

When the VetMAX™ MTBC qPCR kit was applied to a bTB endemic African buffalo population with known skin test and cytokine (IFN-y and IP-10) release assay results for each animal, animals positive for the PCR but with negative culture results were likely to be truly infected (Table 1). This observation was probably due the imperfect sensitivity of mycobacterial culture [11]. Moreover, for skin and/or cytokine assay positive animals, our findings suggest that a validated PCR test may be a better gold standard for determining MTBC infection status when assessing other diagnostic test performance [14].

Furthermore, for all known MTBC-exposed rhinoceros, elephants and buffaloes, the observed 18 PCR-positive, mycobacterial culture-negative results (Table 1), further supports the superior sensitivity of qPCR [11, 12]. These results may also be explained by the presence of residual DNA from dead mycobacteria in these samples, as observed with human TB patients, which would lead to a positive PCR result [22]. However, the culture and PCR negative results for samples from presumed uninfected rhinoceros and elephants also suggest that the VetMAX™ MTBC qPCR Kit may be able to correctly classify uninfected animals as test negative, although this will need to be investigated in future studies.

The discordant results, due to failure of the VetMAX™ MTBC PCR kit to detect DNA in *M. bovis* culture-positive tissue samples (15/57), may be due to DNA degradation, as a result of several freeze-thaw cycles of the stored homogenates [23]. The DNA was extracted from stored tissues that had been previously frozen and thawed, whereas the samples were processed for culture after only one freeze-thaw cycle. However, DNA degradation may have been avoided if homogenized tissue samples, destined for DNA extractions, were stabilized with an appropriate media prior to freezing, or the best option would be to perform DNA extraction on fresh samples.

Based on preliminary data showing that this qPCR can detect MTBC DNA across a range of wildlife species and sample types, this kit appears to be a promising new tool for postmortem TB diagnosis as well as screening antemortem clinical samples. Since this kit can be used across species, it is especially important for wildlife where only limited host-based diagnostic tests are available. It is important to note that the technique appears to be sensitive, although the limitations of this pilot study included limited sample size and freeze-thawing of samples prior to qPCR testing. Additionally, MTBC RT-qPCR test limitations, in general, include the need for qPCR facilities, the detection of genomic targets present in all MTBC members and not individual isolates like *M. bovis* or *M. tuberculosis,* and the high costs related to using qPCRs for population level surveillance programs, may preclude its widespread application.

## Conclusions

Our findings support the use of the VetMAX™ MTBC PCR Kit for the successful amplification of the insertion element IS*6110* from mycobacterial DNA isolated from tissue and respiratory tract samples obtained from *M. bovis*-infected African buffaloes, *M. bovis*-infected white rhinoceros, and *M. tuberculosis-* and *M. bovis*-infected African elephants. Furthermore, this qPCR could provide a new tool for the detection of TB in various sample types from African wildlife species.

## Methods

### Animals

Archived tissue samples from forty-eight African buffaloes from bTB endemic areas, tested between 2016 and 2018 during a bTB test-and-cull programme, were selected for this study. All buffaloes were tested using the single comparative intradermal tuberculin test (SCITT), two separate interferon-gamma (IFN-γ) release assays (IGRAs) and a QuantiFERON TB Gold In-tube (QFT) IFN-γ induced protein of 10 kDa (IP-10) release assay (IPRA), as previously described [24, 25]. All test-positive (i.e., positive on any test) buffaloes were shot by their respective park managers and transported to the abattoir for further processing. Thereafter, tissue samples were opportunistically collected at necropsy for mycobacterial culture and subsequent DNA extractions as in the case of this study. Number of animals selected for bTB testing were done by park management depending on funding available and not by the authors of this study.

Similarly, in 2018 in KNP, antemortem BALF samples were opportunistically collected from four boma-confined white rhinoceros and postmortem tissue samples from six poached rhinoceros found dead (approximately 4–12 h) on arrival. Trunk wash (TW) and BALF samples were also opportunistically collected from 12 chemically immobilized African elephants that included 11 free-ranging animals from KNP and one zoo elephant (South Africa) suspected of being MTBC-infected. In addition, tissue samples were collected from the same zoo elephant after natural death and opportunistically from an elephant carcass discovered in Kruger National Park. All rhinoceros and elephant tissue and respiratory tract samples were processed for DNA extractions and mycobacterial culture. Rhinoceros and elephant immobilizations for BALF and TWs collection were conducted by veterinarians from KNP Veterinary Wildlife Services (South African National Parks) according to their protocols, animals were carefully monitored during this process and all successfully revived. All animals reported in this study were opportunistically sampled during routine immobilizations and not specifically for this study.

### Sample collection and mycobacterial culture

All animal carcasses included in this study were examined for gross TB lesions. Tissue samples with TB-consistent lesions were collected and stored frozen separately. For animals with no visible lesions, samples of head (mandibular, retropharyngeal) and thoracic (mediastinal and tracheobronchial) lymph nodes were pooled by anatomical site. Endoscopic bronchoalveolar lavages were performed on chemically immobilized rhinoceros and elephants as previously described to collect antemortem samples [26, 27]. Additionally, TW samples were obtained from immobilized elephants by flushing 250 ml of sterile saline into each nostril, separately, elevating and lowering the trunk for approximately 30–40 s and aspirating the fluid into a sterile 500 ml collection chamber. Respiratory samples collected in 50 ml sterile falcon tubes were concentrated by centrifugation at 2000 x g for 30 min and decanting 46 ml of the supernatant. The remaining 4 ml with resuspended pellet were stored at − 20 °C. All tissue and respiratory samples were processed for mycobacterial culture using Mycobacterial Growth Indicator Tubes (MGIT™) and the BACTEC™ MGIT™ 960 Mycobacterial Detection System (Becton Dickinson, Franklin Lakes, NJ, USA), as previously described [24]. All Ziehl-Neelsen positively stained bacterial cultures were genetically speciated by PCR, as previously described [19].

### VetMAX™ MTBC PCR kit

After mechanical lysis of all tissue, BALF, and TW samples, DNA was extracted from each sample using the QIAamp® DNA mini kit (Qiagen, Venlo, Limburg, Netherlands) as prescribed by the commercial VetMAX™ MTBC qPCR kit. Extracted DNA (5 μl) was mixed with 20 μl of reaction mix from the PCR kit and the reactions carried out in duplicate using the CFX96 Touch™ Real-Time PCR Detection System (Bio-Rad Laboratories, Hercules, CA, USA). The PCR reactions were done at 50 °C for 2 min (1 cycle), followed by one cycle at 95 °C for 10 min, 40 cycles of 15 s at 95 °C, and 1 min at 60 °C. The experiment was conducted once due to reagent availability. Results were interpreted as negative (C_t_ > 40), positive (C_t_ < 40) or invalid, following the manufacturer’s recommendations and by comparison with multispecies endogenous sample Internal Positive Control and the qPCR test negative and positive qPCR control amplification results. All samples were de-identified prior to DNA extractions to ensure unbiased interpretation of qPCR results and tested in batches of five to avoid possible cross contaminations.

### Statistical analysis

All positive and negative VetMAX™ qPCR and mycobacterial culture results were reported as proportions of the total number of samples tested. All results for the VetMAX™ qPCR, average Ct values and mycobacterial culture results for each sample tested from individual animals are provided in Supplementary Tables 1 and 2.

## Supplementary information

**Additional file 1: Table S1.** Test results for *n* = 48 African buffaloes (*Syncerus caffer*) randomly selected from two geographically distinct endemic areas and their mycobacterial culture results. **Table S2.** VetMAX PCR Kit test results for tissue and respiratory samples from *n* = 10 white rhinoceros (*Ceratotherium simum*) and *n* = 24 African elephants (*Loxodonta africana*) with known mycobacterial culture results.

## Data Availability

The raw data generated during the current study are available from the corresponding author on reasonable request. Moreover, all results for each animal are available in the accompanying supplementary tables.

## Abbreviations

MTBC: *Mycobacterium tuberculosis* complex
*M. bovis*: *Mycobacterium bovis*
*M. tuberculosis*: *Mycobacterium tuberculosis*
bTB: Bovine tuberculosis
TB: Tuberculosis
KNP: Kruger National Park
CMI: Cell-mediated immunity
BALF: Bronchoalveolar lavage fluid
TW: Trunk wash
RT-qPCR: Real time quantitative polymerase chain reaction
IS*6110*: Insertion element 6110
IFN-γ: Interferon gamma
IP-10: Interferon gamma-induced protein of 10 kDa
SCITT: Single comparative intradermal tuberculin test
IGRA: IFN-γ release assay
IPRA: IP-10 release assay
QFT: QuantiFERON^®^ TB Gold In-tube assay
MGIT: Mycobacterial growth indicator tuber
ZN stains: Ziehl-Neelsen staining
